# Dynamic changes in prognostic nutritional index and systemic immune-inflammation index predict survival following debulking surgery in ovarian cancer

**DOI:** 10.3389/fonc.2025.1720075

**Published:** 2026-01-13

**Authors:** Karolin Ohanoglu Cetinel, Gazi Guner, Can Berk Karabudak, Emilya Zeynalli, Suheda Yavuz Sen

**Affiliations:** 1 Basaksehir Cam and Sakura City Hospital Department of Obstetrics and Gynecology, Istanbul, Türkiye; 2 Basaksehir Cam and Sakura City Hospital Department of Gynecologic Oncology, Istanbul, Türkiye

**Keywords:** debulking surgery, ovarian cancer, overall survival, prognostic nutritional index, systemic immune-inflammation index

## Abstract

**Background:**

Ovarian cancer remains the most lethal gynecologic malignancy. Increasing evidence suggests that host immunonutritional status and systemic inflammation—captured by the Prognostic Nutritional Index (PNI) and the Systemic Immune-Inflammation Index (SII)—influence postoperative recovery and survival outcomes.

**Methods:**

This retrospective study included 78 patients who underwent primary debulking surgery for epithelial ovarian cancer. Preoperative and 6-hour postoperative PNI and SII values were calculated, and ΔPNI was defined as the postoperative–preoperative difference. Associations with postoperative inflammatory markers and overall survival (OS) were evaluated using Spearman correlation, Kaplan–Meier analysis, and Cox regression.

**Results:**

Postoperative PNI decreased significantly (49.3 → 34.1; p < 0.001), and SII increased markedly (964.7 → 4003.2; p < 0.001). Lower postoperative PNI and higher preoperative SII were associated with greater postoperative inflammatory response. In multivariate analysis, higher postoperative PNI independently predicted improved OS (HR 0.94, 95% CI 0.89–0.99; p = 0.021), while higher preoperative SII independently predicted worse OS (HR 1.18, 95% CI 1.01–1.36; p = 0.037). ΔPNI and postoperative SII were not independently prognostic.

**Conclusion:**

Postoperative PNI and preoperative SII provide complementary and independent prognostic information in patients undergoing primary debulking surgery for ovarian cancer. ΔPNI reflects acute immunonutritional stress but does not independently predict survival. Given their accessibility and modifiability, these indices may support perioperative risk stratification and represent potential targets for future interventional studies.

## Introduction

Ovarian cancer remains the most lethal gynecologic malignancy worldwide, largely because it is often diagnosed at advanced stages when curative treatment is limited. Optimal cytoreductive surgery and platinum-based chemotherapy remain the cornerstones of management, yet survival outcomes vary considerably even among patients receiving standard care. Increasing evidence suggests that host-related factors, particularly nutritional and inflammatory status, play a critical role in shaping prognosis.

The Prognostic Nutritional Index (PNI)—calculated from serum albumin and lymphocyte count—serves as a surrogate of nutritional reserve and immune competence. Low PNI has been consistently associated with inferior survival outcomes across malignancies, including ovarian cancer. A recent meta-analysis demonstrated that reduced preoperative PNI correlates with shorter overall survival (OS) in ovarian cancer patients (1). Yoshikawa et al. reported that in early-stage clear cell carcinoma, PNI <46.5 was independently predictive of poor survival (2), while Rubio-Cordero et al. confirmed the prognostic utility of PNI across epithelial subtypes (3).

In parallel, systemic inflammation is increasingly recognized as a driver of tumor progression and immune evasion. The Systemic Immune-Inflammation Index (SII), calculated as platelet × neutrophil/lymphocyte counts, integrates key inflammatory and immune cellular components. Elevated SII has been linked to worse outcomes in multiple malignancies. For example, a meta-analysis of patients receiving immune checkpoint inhibitors showed that high SII predicted shorter Overall Survival (OS) across tumor types (4), and Jomrich et al. identified SII as an independent survival predictor in pancreatic ductal adenocarcinoma, outperforming platelet-to-lymphocyte ratio (PLR) and neutrophil-to-lymphocyte ratio (NLR) (5).

Despite the growing body of evidence, few studies have examined perioperative changes in PNI (ΔPNI) alongside SII in the context of primary debulking surgery for ovarian cancer. Dynamic perioperative shifts in PNI may better capture the physiologic impact of surgical stress and evolving immunonutritional status, while SII could provide complementary insights into systemic inflammatory burden.

The present study aimed to investigate the prognostic role of preoperative and postoperative PNI, perioperative ΔPNI, and SII in ovarian cancer patients undergoing primary debulking surgery. Specifically, we sought to: I) evaluate their associations with postoperative inflammatory markers (CRP kinetics, lactate, pH) and complications (intensive care unit (ICU) stay, total parenteral nutrition (TPN), octreotide use), II) explore the interrelationship between PNI and SII, and III) compare the predictive value of PNI and SII for overall survival in a multivariable framework. To our knowledge, this is the first study to assess both perioperative changes in PNI and SII in this patient population.

## Methods

This retrospective observational study was carried out in the Department of Gynecologic Oncology at Başakşehir Çam and Sakura City Hospital, Istanbul, Türkiye. The study population consisted of patients with histologically confirmed epithelial ovarian cancer who underwent primary debulking surgery performed by the same surgical team between January 2020 and December 2025. A total of 120 consecutive patients were initially screened, and after applying the eligibility criteria, 78 were included in the final analysis.

Inclusion criteria consisted of 1) histopathologically confirmed epithelial ovarian carcinoma, 2) primary cytoreductive surgery performed as the first-line surgical intervention, 3) availability of both preoperative and postoperative laboratory data (including serum albumin, lymphocyte, neutrophil, and platelet counts), and 4) complete follow-up and survival information.

Exclusion criteria included: 1) concurrent or previous endometrial carcinoma, 2) interval or secondary cytoreductive surgery following neoadjuvant chemotherapy, and 3) missing or incomplete laboratory or clinical records. In addition, to minimize confounding effects on immunonutritional and inflammatory markers, patients with active primary malignancies other than ovarian cancer and those with chronic systemic inflammatory, metabolic, or autoimmune diseases known to significantly influence PNI or SII (such as cirrhosis, advanced chronic kidney disease, chronic obstructive pulmonary disease, or autoimmune disorders) were intentionally excluded, although such patients may undergo cytoreductive surgery at our institution. This ensured that the perioperative changes in PNI and SII reflected the physiological response to ovarian cancer surgery rather than unrelated comorbid conditions. Importantly, none of the included patients had documented postoperative infectious complications during the perioperative period.

All surgical procedures were performed by the same gynecologic oncology team to minimize inter-operator variability. The extent of cytoreduction followed institutional and NCCN guideline–based protocols, aiming for no macroscopic residual disease. Residual disease status was documented intraoperatively and classified according to standard gynecologic oncology criteria as R0 (no macroscopic residual disease), R1 (residual disease ≤1 cm), or R2 (residual disease >1 cm). Patients received standardized perioperative care and postoperative monitoring within the same institution to ensure uniform management.

All patients underwent preoperative anesthetic and laboratory evaluation within seven days before surgery to obtain baseline hematologic and biochemical parameters. Postoperative blood samples were collected 6 hours after completion of surgery in accordance with our institutional monitoring protocol. This standardized early timepoint was selected to minimize the confounding effects of intravascular fluid shifts, hemodilution from perioperative fluid administration, diurnal variation, and interventions occurring during early ward or ICU care. Previous studies assessing perioperative albumin dynamics, lymphocyte responses, and inflammatory markers have similarly used 6–12 hour windows to capture the immediate physiologic impact of surgical stress.

Venous blood gas measurements, including pH and lactate, were obtained during the same 6-hour postoperative window. In addition, CRP values were routinely measured on postoperative days 1, 3, and 5 as part of the institutional infection surveillance protocol, providing a standardized assessment of early postoperative inflammatory response and its potential relationship to immunonutritional indices.

Postoperative management was conducted following a uniform institutional protocol. Patients were monitored in the recovery room and transferred either to the gynecologic oncology ward or the ICU, depending on hemodynamic stability and intraoperative blood loss. Administration of TPN and octreotide was recorded, as these interventions could influence nutritional or inflammatory parameters. Blood transfusions were administered based on a standardized threshold (hemoglobin <8 g/dL or symptomatic anemia).

Fluid balance, antibiotic prophylaxis (single-dose cefazolin within 30 minutes before incision), and postoperative analgesia were applied homogeneously across all cases. No patient received corticosteroids or immunosuppressive therapy during the perioperative period.

All blood samples were processed in the hospital’s central biochemistry and hematology laboratories, accredited under ISO 15189 standards. Serum albumin levels were measured using the bromocresol green method on a Beckman Coulter AU5800 analyzer. Complete blood counts were performed using an automated hematology analyzer (Sysmex XN-1000, Kobe, Japan). Internal and external quality controls were performed daily to ensure consistency and reproducibility.

The Prognostic Nutritional Index (PNI) was calculated as (6):


$$\text{PNI }=\;\left(10\;\times \text{ serum albumin }\left[\text{g}/\text{dL}\right]\right)\;+\;\left(0.005\;\times \text{ lymphocyte count }\left[/{\text{mm}}^{3}\right]\right)$$

Both preoperative and postoperative PNI values were determined. The change in PNI (ΔPNI) was calculated as ΔPNI= PNI_post_− PNI_pre_ to quantify perioperative fluctuations in nutritional and immune status.

The Systemic Immune-Inflammation Index (SII) was computed using the formula (7):


SII=Plt (N/L) (platelet count)×(neutrphil count /lymphocyte  count)

Preoperative and postoperative SII values were obtained for each patient. This index has been validated as a reliable indicator of the balance between inflammation and immune response in several malignancies and was used as a complementary marker to PNI in the present study.

The primary endpoint was OS, defined as the time from the date of surgery to death from any cause or last known follow-up. Survival status and duration (in years) were confirmed via hospital records and the national death registry. Secondary outcomes included correlations between PNI, ΔPNI, SII, and postoperative inflammatory markers such as CRP, lactate, and pH, as well as associations with clinical outcomes (ICU stay, TPN administration, octreotide use).

### Ethical considerations

The study protocol was approved by the Institutional Review Board/Ethics Committee of Başakşehir Çam and Sakura City Hospital (Decision date/number: 05.03.2025/86). Owing to the retrospective design and the use of anonymized data, informed consent was waived. All procedures were conducted in accordance with the Declaration of Helsinki and the Good Clinical Practice (GCP) guidelines.

### Statistical analysis

All analyses were performed using SPSS version 29.0 (IBM Corp., Armonk, NY, USA) and Python (for supplementary survival analyses and figure standardization). Data normality was assessed using the Kolmogorov–Smirnov and Shapiro–Wilk tests. Normally distributed variables were expressed as mean ± standard deviation (SD), and non-normally distributed variables as median (interquartile range, IQR). Categorical variables were summarized as counts and percentages.

Between-group comparisons were performed using the independent-samples t-test or Mann–Whitney U test for continuous variables and the Chi-square test or Fisher’s exact test for categorical variables. Correlations between perioperative immunonutritional indices (PNI, ΔPNI, SII) and postoperative inflammatory parameters (CRP, lactate, pH) were evaluated using Spearman’s rank correlation.

SII demonstrated a right-skewed distribution and was therefore log10-transformed before its inclusion in Cox regression models. Overall survival (OS) was estimated using the Kaplan–Meier method, with differences assessed by the log-rank test. For visualization, PNI and SII were dichotomized using median cutoffs because the limited number of events precluded reliable ROC-based threshold determination; continuous-variable Cox models were used to avoid information loss.

Univariate Cox proportional hazards regression analyses were conducted for each variable separately. Variables with p < 0.10 in univariate analysis were entered into the multivariate Cox model to identify independent prognostic factors. Hazard ratios (HR) with 95% confidence intervals (CI) were calculated, and a two-sided p < 0.05 was considered statistically significant.

## Results

A total of 78 patients with histologically confirmed ovarian cancer who underwent primary debulking surgery were included. The median age was 56.5 years (IQR 16.8). Among the 78 patients included, the most common histological subtype was serous carcinoma (60.3%), followed by endometrioid (20.5%), clear cell carcinoma (5.1%), and other histologies (14.1%). The majority of patients presented with advanced disease, with FIGO stage 4A (26.9%) and stage 4B (19.2%) being the most frequent. Early-stage cases (stage I–II) accounted for less than one-third of the cohort. The median follow-up duration among surviving patients was 2.0 years (IQR 2.0–3.0). At last follow-up, 22 of 78 patients (28.2%) had died of disease.

The mean preoperative PNI was 49.3 ± 7.0, significantly decreasing to 34.1 ± 7.8 postoperatively (p < 0.001). Similarly, the mean SII increased markedly from 964.7 ± 892.4 preoperatively to 4003.2 ± 4272.7 postoperatively (p < 0.001), reflecting the expected postoperative inflammatory surge. These perioperative immunonutritional changes are summarized in Section A of **Table 1**.

**Table 1:** Perioperative immunonutritional, inflammatory, and metabolic parameters.

A. Perioperative changes in PNI and SII				
Marker	Preoperative (mean ± SD)	Postoperative (mean ± SD)	Median change (Δ, IQR)	P-value
PNI	49.3 ± 7.0	34.1 ± 7.8	−15.8 (11.1)	<0.001
SII	964.7 ± 892.4	4003.2 ± 4272.7	+2988 (3399.9)	<0.001

B. Inflammatory and metabolic parameters (Median, IQR)	
Marker	Median (IQR)
CRP Day 1 (mg/L)	51.6 (12.3–145.1)
CRP Day 3 (mg/L)	93.9 (48.4–151.8)
CRP Day 5 (mg/L)	161.8 (65.7–228.8)
Lactate (mmol/L)	1.4 (0.9–2.6)
pH	7.39 (7.32–7.46)

PNI = Prognostic Nutritional Index; SII = Systemic Immune-Inflammation Index. Values in section A are presented as mean ± standard deviation, with median (interquartile range) shown for perioperative change (Δ). Values in section B are presented as median (interquartile range). CRP values were obtained on postoperative days 1, 3, and 5. Lactate and pH were measured 6 hours after surgery as part of standardized postoperative monitoring.

Distributions of postoperative inflammatory and metabolic parameters—including CRP (days 1, 3, and 5), lactate, and pH—are presented as median values with interquartile ranges in Section B of **Table 1**, providing a complementary overview of early postoperative inflammatory and metabolic responses.

Values are presented as median (interquartile range). CRP values represent postoperative measurements obtained on days 1, 3, and 5. Lactate and pH were measured 6 hours after completion of surgery as part of the standardized postoperative monitoring protocol.

As shown in **Table 2**, both preoperative and postoperative PNI correlated negatively with CRP on postoperative day 5 (ρ = –0.27, p = 0.020), and postoperative PNI also correlated inversely with lactate (ρ = –0.31, p = 0.007). In contrast, preoperative SII correlated positively with CRP day 5 (ρ = 0.33, p = 0.004) and lactate (ρ = 0.29, p = 0.009). Postoperative SII showed no significant correlation with CRP, lactate, or pH. No meaningful correlations were observed between PNI or SII values and blood pH, indicating that perioperative nutritional and inflammatory indices were more closely related to inflammatory and metabolic markers than to acid–base balance.

**Table 2:** Correlations of perioperative PNI and SII with inflammatory and metabolic parameters.

Inflammatory and metabolic parameters	Preop PNI (ρ, p)	Postop PNI (ρ, p)	Preop SII (ρ, p)	Postop SII (ρ, p)
CRP day 1	0.08, 0.49	0.15, 0.21	0.18, 0.13	0.11, 0.34
CRP day 3	0.09, 0.45	−0.02, 0.84	0.21, 0.08	0.13, 0.26
CRP day 5	−0.27, 0.020*	−0.27, 0.020*	0.33, 0.004*	0.09, 0.42
Lactate	−0.18, 0.12	−0.31, 0.007*	0.29, 0.009*	0.12, 0.28
pH	0.04, 0.74	0.03, 0.80	−0.05, 0.67	−0.08, 0.51

*Values represent Spearman’s correlation coefficient (ρ) and p-values.

**Statistically significant correlations (p < 0.05).

PNI, Prognostic Nutritional Index; SII, Systemic Immune-Inflammation Index; CRP, C-reactive protein.

As shown in **Table 3**, preoperative PNI correlated negatively with preoperative SII (ρ = −0.43, p < 0.001), indicating that lower nutritional and immune reserve was associated with higher systemic inflammation. A similar inverse association was found between postoperative PNI and preoperative SII (ρ = −0.31, p = 0.005). No significant relationship was observed between postoperative PNI and postoperative SII. (**Table 3**).

**Table 3:** Correlations between PNI and SII.

Comparison	Spearman ρ	P-value
Preop PNI vs Preop SII	−0.43	<0.001
Postop PNI vs Preop SII	−0.31	0.005
Postop PNI vs Postop SII	0.06	0.62

*PNI = Prognostic Nutritional Index; SII = Systemic Immune-Inflammation Index; Preop = preoperative; Postop = postoperative.

Multivariate Cox analysis was performed to identify independent prognostic factors for overall survival (**Table 4**). After adjustment for age, FIGO stage, and histology, postoperative PNI remained an independent protective factor (HR 0.94, 95% CI 0.89–0.99, p = 0.021), indicating that higher postoperative PNI was associated with improved survival. In contrast, preoperative SII independently predicted worse survival (HR 1.18, 95% CI 1.01–1.36, p = 0.037). Preoperative PNI and postoperative SII were not significant predictors in the adjusted model. Among clinical covariates, advanced FIGO stage (III–IV) was significantly associated with reduced survival (HR 1.67, p = 0.015).

**Table 4:** Multivariate Cox regression for overall survival.

Variables	HR	95% CI	P-value
Postoperative PNI (continuous)	0.94	0.89–0.99	0.021**
ΔPNI (post – pre)	1.02	0.99–1.05	0.11
Preoperative PNI	0.98	0.94–1.02	0.28
Preoperative SII (log-transformed)	1.18	1.01–1.36	0.037**
Postoperative SII (log-transformed)	1.05	0.92–1.19	0.42
FIGO Stage (III–IV vs I–II)	1.67	1.12–2.49	0.015**
Age (per 10 years)	1.12	0.95–1.31	0.18
Histology (serous vs others)	1.03	0.72–1.47	0.84

*HR = hazard ratio; CI = confidence interval; PNI = Prognostic Nutritional Index; SII = Systemic Immune-Inflammation Index.

ΔPNI indicates postoperative change (postoperative − preoperative).

**p < 0.05, statistically significant.

In univariate Cox regression analysis several perioperative immunonutritional and clinical variables showed significant associations with overall survival. Higher preoperative PNI and greater postoperative decreases in PNI (ΔPNI) were both significantly associated with poorer survival. Preoperative and postoperative SII (log-transformed), age, and advanced FIGO stage (III–IV) also demonstrated strong adverse prognostic effects in univariate models. In contrast, postoperative PNI and histologic subtype did not show significant associations with survival in univariate analysis (**Table 5**).

**Table 5:** Univariate Cox regression analysis for overall survival.

Variable	HR	95% CI (lower–upper)	P-value
Preoperative PNI (continuous)	0.96	NA	<0.001
Postoperative PNI (continuous)	0.97	0.92–1.03	0.317
ΔPNI (post – pre)	1.01	NA	<0.001
Preoperative SII (log10-transformed)	0.68	NA	<0.001
Postoperative SII (log10-transformed)	0.57	NA	<0.001
Age (per 10 years)	1.22	NA	<0.001
FIGO stage (III–IV vs I–II)	4.08	NA	<0.001
Histology (serous vs others)	1.18	0.51–2.78	0.697

HR, hazard ratio; CI, confidence interval; PNI, Prognostic Nutritional Index; SII, Systemic Immune-Inflammation Index; NA, Not Available. SII values were log10-transformed before analysis. For some variables, confidence intervals could not be estimated reliably due to model instability and the limited number of events; therefore, only hazard ratios and p-values are presented.

**Figure 1:**
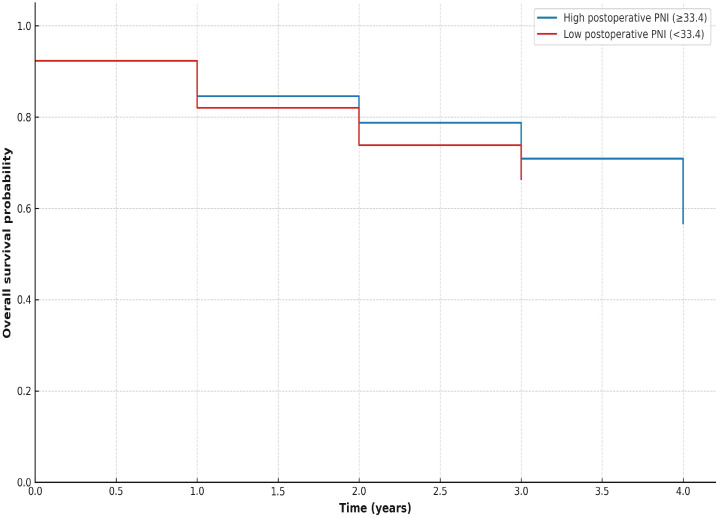
Kaplan–Meier curves showing overall survival according to postoperative PNI (median cutoff). Patients with low postoperative PNI exhibited shorter overall survival compared with those with high postoperative PNI.

**Figure 2:**
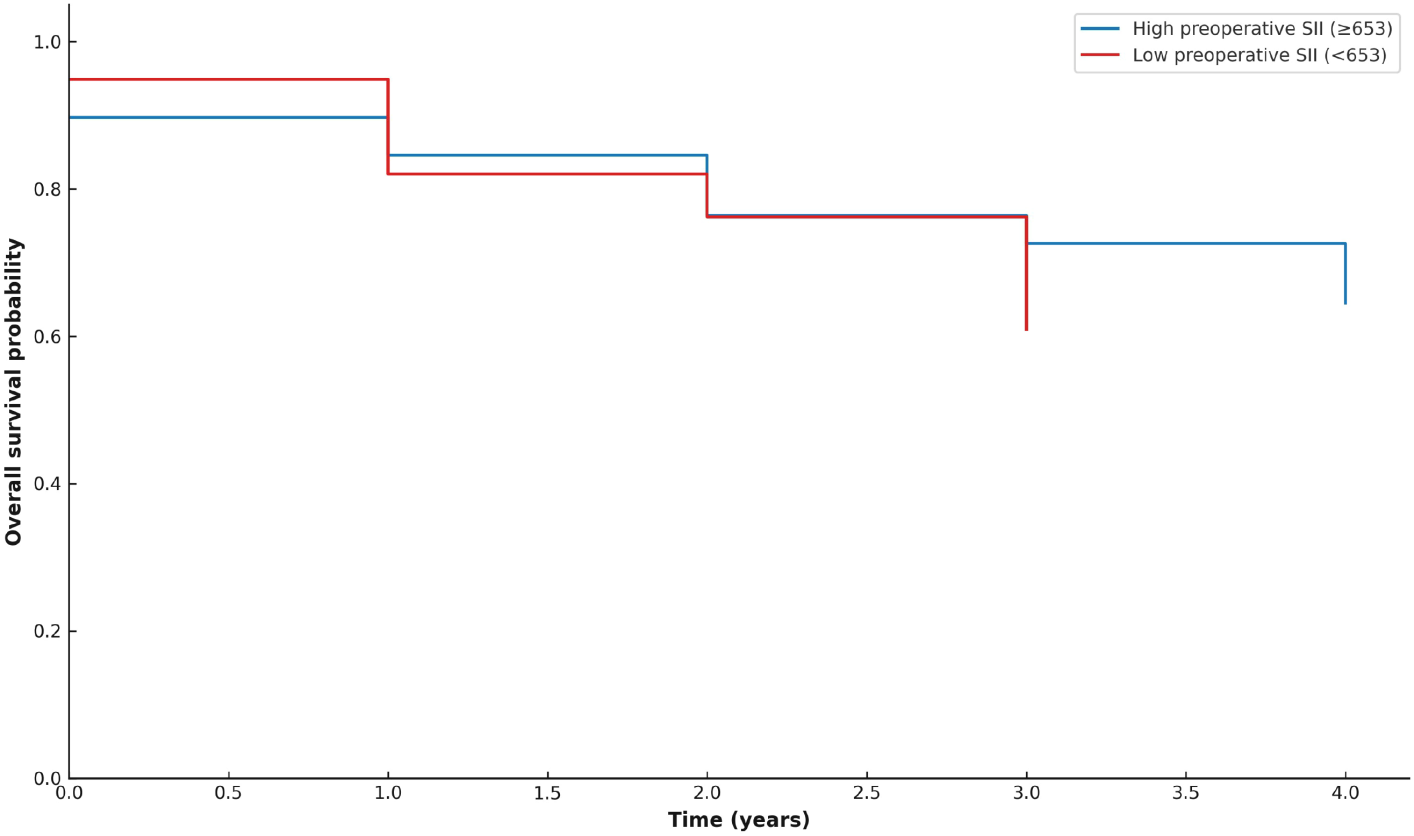
Kaplan–Meier curves showing overall survival according to preoperative SII (median cutoff). Survival curves overlapped substantially between high and low SII groups.

Kaplan–Meier survival curves were generated to visualize overall survival according to postoperative PNI and preoperative SII categories (median-based grouping). Patients with low postoperative PNI (<33.4) exhibited shorter overall survival compared with those with high postoperative PNI (≥33.4) (**Figure 1**). Patients with high preoperative SII (≥653, red line) and those with low SII (<653) showed largely overlapping survival curves, indicating no significant difference in unadjusted survival (**Figure 2**). However, in multivariate Cox regression, higher preoperative SII independently predicted poorer overall survival (HR 1.18, 95% CI 1.01–1.36, p = 0.037).

Postoperative supportive care requirements were also evaluated. ICU admission occurred in 14.1% of patients and was not significantly associated with either pre- or postoperative PNI or SII. Total parenteral nutrition (TPN) was administered in 10.3% of patients, exclusively in the low postoperative PNI group, although this difference did not reach statistical significance (p = 0.115). Similarly, octreotide use was more frequent among patients with low postoperative PNI (28.2% vs. 15.4%), without reaching statistical significance (p = 0.273). No significant associations were observed between SII values and any of these postoperative interventions (**Table 6**).

**Table 6:** Postoperative supportive care requirements according to postoperative PNI status.

Postoperative supportive care variables	Low PNI (n, %)	High PNI (n, %)	P-value
ICU admission	22/39 (56.4%)	16/39 (41.0%)	0.257
TPN use	4/39 (10.3%)	0/39 (0.0%)	0.115
Octreotide use	11/39 (28.2%)	6/39 (15.4%)	0.273

Groups defined by median postoperative PNI cutoff.

p-values from chi-square or Fisher’s exact test, as appropriate.

A review of postoperative records also demonstrated no major complications (Clavien–Dindo grade ≥3) in this cohort. Minor complications were limited to transient postoperative ileus and electrolyte imbalance, all of which were managed conservatively and showed no significant differences between groups.

Among the 78 patients, 88% achieved R0 cytoreduction, 8% had R1 disease, and 4% had R2 disease. Across the R0–R1–R2 groups, no statistically significant differences were observed in preoperative PNI, postoperative PNI, ΔPNI, or preoperative/postoperative SII values (all p > 0.05). Patients with R2 cytoreduction showed numerically lower postoperative PNI and higher preoperative SII, but these trends did not reach statistical significance, likely due to the small number of R1–R2 cases (**Table 7**).

**Table 7:** Association between cytoreduction completeness (R0–R1–R2) and perioperative immunonutritional indices (PNI, ΔPNI, SII).

Residual disease status	n (%)	Preoperative PNI (mean ± SD)	Postoperative PNI (mean ± SD)	ΔPNI (post–pre)	Preoperative SII (mean ± SD)	Postoperative SII (mean ± SD)	P-value
R0	69 (88%)	49.6 ± 6.8	34.5 ± 7.6	–15.1 ± 6.3	942.1 ± 861.4	3912.6 ± 4175.2	>0.05
R1	6 (8%)	47.8 ± 7.5	32.1 ± 8.4	–15.7 ± 7.1	1185.3 ± 1042.8	4297.4 ± 4520.1	>0.05
R2	3 (4%)	45.3 ± 8.1	29.4 ± 9.2	–15.9 ± 8.0	1348.6 ± 1205.3	4563.9 ± 4988.7	>0.05
Overall	78	49.3 ± 7.0	34.1 ± 7.8	–15.2 ± 6.6	964.7 ± 892.4	4003.2 ± 4272.7	—

PNI = Prognostic Nutritional Index; ΔPNI = change in PNI (postoperative – preoperative); SII = Systemic Immune-Inflammation Index. p-values represent comparisons across R0–R1–R2 groups; none reached statistical significance.

## Discussion

In this cohort of ovarian cancer patients undergoing primary debulking surgery, we demonstrated that postoperative PNI and preoperative SII are prognostically relevant for overall survival, while preoperative PNI and postoperative SII showed no independent association. Although ΔPNI did not independently predict survival, its decline reflects the immunonutritional impact of surgical stress and may have incremental value when interpreted alongside postoperative PNI. Patients with poorer perioperative nutritional and immune status appear to experience greater inflammatory and metabolic stress following debulking surgery. The inverse association between PNI and CRP or lactate supports the ESPEN guidelines concept that malnutrition aggravates surgical inflammation and delays recovery (8). Combined assessment of immunonutritional and inflammatory indices may refine perioperative risk stratification and guide individualized management. Although ΔPNI did not remain independently prognostic in multivariate analysis, this may reflect overlap with postoperative PNI, the early 6-hour postoperative sampling time, and the limited number of survival events.

In multivariate analysis, postoperative PNI emerged as an independent protective factor for overall survival, while preoperative SII retained independent adverse prognostic significance in multivariate analysis. Although Kaplan–Meier curves for SII groups were largely overlapping and the difference was not significant in the unadjusted analysis, the direction of the effect was consistent with the multivariate model, suggesting an independent adverse impact of systemic inflammation. These findings indicate that distinct immunonutritional and inflammatory mechanisms exert prognostic influence at different perioperative time points: elevated preoperative SII identifies patients with baseline systemic inflammation, whereas higher postoperative PNI reflects favorable nutritional recovery and improved host resilience. This discrepancy between Kaplan–Meier curves and multivariate Cox results suggests confounding by stage or age in the unadjusted analysis, with the independent adverse effect of systemic inflammation becoming clear only after adjustment. Recent studies have also demonstrated that systemic inflammatory indices, including SII, carry prognostic value even in early-stage ovarian cancer, supporting the broader applicability of our findings (9, 10).

Our results align with prior studies demonstrating the prognostic value of PNI in ovarian and other malignancies. Zhang et al. first reported that preoperative PNI predicted platinum resistance and survival in ovarian cancer (11), later confirmed by Tan et al. in a larger cohort (12). Liu et al. also showed that preoperative immunonutritional status, including PNI, independently predicted outcomes (13). Beyond ovarian cancer, meta-analyses in gastrointestinal and gynecologic malignancies have reinforced the prognostic value of PNI, linking low PNI with adverse tumor features and poor survival (14, 15). Similarly, SII has been validated as a robust prognostic marker across multiple tumor types. Jomrich et al. found SII superior to NLR and PLR in pancreatic ductal adenocarcinoma (5), and Hu et al. confirmed its prognostic value in hepatocellular carcinoma (7). In ovarian cancer, Mao and Yang demonstrated in a meta-analysis that high SII was associated with shorter OS and PFS (Progression-Free Survival) (16), while Chu et al. confirmed these results in an updated meta-analysis (17). Chen et al. reported that combining PNI and SII improved prognostic accuracy in epithelial ovarian cancer (18), aand other studies have demonstrated that PNI, together with indices such as PLR and HALP, predicts survival after primary debulking (19). Further work has supported integrating multiple inflammatory and nutritional markers for risk stratification in ovarian cancer (20).

Residual tumor burden is an important prognostic factor in ovarian cancer. In our cohort, 88% of patients achieved R0 cytoreduction, and although residual disease status was not significantly associated with perioperative PNI or SII, patients with R1–R2 cytoreduction showed a consistent trend toward lower postoperative PNI and higher preoperative SII. This pattern is clinically plausible and is consistent with prior literature linking greater tumor burden with heightened systemic inflammation and impaired immunonutritional status (21).

The biological plausibility of these associations is strong. PNI reflects both nutritional reserve and immune competence, whereas SII integrates the pro-tumor roles of neutrophils and platelets with the anti-tumor role of lymphocytes. Hypoalbuminemia and lymphopenia indicate catabolic stress and impaired immunity, while elevated SII represents a pro-tumor inflammatory milieu promoting angiogenesis, immune evasion, and metastasis (22). Labelle and Hynes described how neutrophil–platelet interactions facilitate early metastatic events (23), supporting the mechanistic rationale for our findings.

Clinically, these results underscore the utility of simple, low-cost laboratory indices in perioperative monitoring. Incorporating ΔPNI and preoperative SII into clinical practice could allow early identification of high-risk patients, prompting closer postoperative surveillance or targeted nutritional optimization. The ESPEN surgical guidelines emphasize that perioperative nutritional support improves outcomes in high-risk patients (8), and our data support integrating such strategies with inflammatory modulation in those exhibiting persistently high SII. Ultimately, combined assessment of PNI and SII may enhance prognostic modeling and guide personalized postoperative management. Incorporating postoperative PNI and preoperative SII into perioperative assessment may therefore help identify high-risk patients who could benefit from intensified monitoring or targeted nutritional and anti-inflammatory optimization.

Strengths of this study include the availability of both pre- and postoperative laboratory data, enabling ΔPNI calculation, and the homogeneity of a cohort restricted to primary debulking cases. To our knowledge, this is the first study to evaluate perioperative ΔPNI and SII concurrently in this population.

## Limitations

This study has several limitations. Its retrospective, single-center design and modest sample size may limit generalizability, although all consecutive eligible patients were included to minimize selection bias. Detailed documentation of chronic inflammatory comorbidities and perioperative nutritional interventions was limited by the retrospective design. The use of 6-hour postoperative laboratory values reflects institutional practice and aligns with previous perioperative studies, although additional time points (e.g., 24–48 hours) may provide further insight into delayed inflammatory or nutritional changes. Although the study was conducted within a 5-year follow-up framework, the median observed follow-up among survivors was 2.0 years, limiting long-term survival interpretation. The limited number of survival events also precluded reliable ROC-based cutoff determination; therefore, PNI and SII were analyzed primarily as continuous variables, with median cutoffs used only for Kaplan–Meier visualization. Larger multicenter prospective studies are needed to validate these findings and assess whether perioperative immunonutritional optimization can improve oncologic outcomes.

## Conclusion

Postoperative PNI and preoperative SII provide complementary prognostic information in ovarian cancer patients undergoing primary debulking surgery. ΔPNI reflects acute perioperative immunonutritional stress, while postoperative PNI and preoperative SII identify patients with impaired recovery or heightened systemic inflammation. Combined assessment of these simple, widely available indices may improve perioperative risk stratification and support personalized postoperative management. Prospective studies should evaluate whether interventions targeting nutritional and inflammatory pathways can modify these biomarkers and improve survival.

## Data Availability

The original contributions presented in the study are included in the article/supplementary material. Further inquiries can be directed to the corresponding author.
